# The impact of alumni football on adult mental wellbeing: a serial mediation analysis of athletic identity and social connectedness

**DOI:** 10.3389/fpsyg.2026.1780689

**Published:** 2026-03-10

**Authors:** Jiao Wang

**Affiliations:** 1 Faculty of Artificial Intelligence in Education, Central China Normal University, Wuhan, Hubei, China; 2 School of Physical Education, Hanjiang Normal University, Shiyan, Hubei, China

**Keywords:** alumni football, athletic identity, mental wellbeing, serial mediation, social connectedness

## Abstract

**Background:**

Escalating life pressures among adults are increasingly associated with social isolation. Distinct from general team sports, “Alumni Football” facilitates a unique dual-mechanism of identity restoration and social reconnection, particularly for adults navigating the loss of former athletic roles. However, the specific pathways through which re-established athletic identity relates to social connectedness and correlates with wellbeing remain under-explored.

**Methods:**

This study employs a cross-sectional design involving 240 alumni football participants. Using Structural Equation Modeling (SEM), it systematically analyzes the structural relationships between participation, athletic identity, social connectedness, and mental wellbeing.

**Results:**

The data analysis delineates a specific chain mediation path: actual participation is positively associated with athletic identity, which is linked to social connectedness, ultimately correlating with mental wellbeing. Notably, while athletic identity serves as a crucial link, it exhibits no significant direct association with mental wellbeing (β = 0.05). This finding implies that athletic identity alone, lacking the support of social connectedness, may be insufficient to relate to improved psychological health during the transition out of competitive contexts.

**Conclusion:**

Findings underscore the positive relationship between alumni football and adult mental wellbeing, highlighting social connectedness as the core mediator. The study suggests leveraging alumni networks for mental wellbeing interventions, advocating a strategic pivot from merely emphasizing “exercise volume” to cultivating identity-based “sporting communities” to potentially mitigate social isolation risks.

## 1 Introduction

Adults today face escalating psychological challenges, including burnout (Dilekçi et al., 2025), life stress (Zhang et al., 2025), and alienation (Jiang et al., 2025). Addressing these burdens has become a public health priority, shifting focus toward lifestyle-based interventions (Legg et al., 2025). While physical activity is a known remedy for distress (Stone et al., 2023; Luo et al., 2023), team sports offer distinct advantages over individual exercise. Their interactive nature and provision of group support make them particularly effective for improving anxiety levels and subjective wellbeing (Grunberg et al., 2024; Eather et al., 2023).

With its immense global appeal, football functions as a vital conduit for social interaction (McKeown et al., 2015; Wei et al., 2025). Beyond a mere test of individual skill, the sport necessitates a fusion of tactical synergy and emotional resonance, creating fertile ground for deep friendships. However, adults frequently struggle to sustain these bonds due to geographical dispersion and the high logistical costs of organizing teams (Lange et al., 2025). “Alumni football” emerges as a solution to this disconnect, seamlessly integrating sport with the pre-existing social ties of a shared alma mater (Wang, 2025). Unlike general community sports or commercial leagues which often rely on transient interactions among strangers, alumni football distinguishes itself by leveraging pre-established social capital. By interlocking the interactivity of football with the inherent institutional trust of alumni networks, it constructs a unique “psychologically safe space”. Here, participants reconnect not only with former teammates but also with their past selves (Nathan et al., 2013), effectively bridging the gap between their current adult lives and their former athletic identities.

Central to this dynamic is the construct of “Athletic Identity” (Mitchell et al., 2014; Edison et al., 2021). Drawing on self-perception theory, which posits that individuals infer their roles through the observation of their own behaviors (Bem, 1972; Bortoli et al., 2012), participation in alumni football transcends mere recreation for adults; it serves as a process of consolidating self-concept (Öztaş and Vural, 2025). However, existing literature also warns of the potential downsides of strong athletic identification, such as “identity foreclosure”, where an exclusive commitment to the athlete role can lead to distress during the transition out of competitive sport or in the face of injury (Britton W Brewer and Petitpas, 2017). When this primary source of self-worth is removed, individuals with a rigid athletic identity are prone to severe psychological distress, including depression, anxiety, and a profound loss of meaning in life (Gorman and Blackwood, 2025). Therefore, while the temporary regression from “daily roles” to that of an “athlete” affords participants a renewed sense of mastery and self-worth, it is critical to understand whether this identity directly supports wellbeing or if it requires a social conduit to prevent the isolation often associated with identity rigidity.

To mitigate these potential downsides, identity recognition must be externalized as social connectedness to maximize mental wellbeing benefits (McNamara et al., 2021). Theoretically, this aligns with Social Identity Theory, which suggests that shared group membership provides the necessary psychological scaffolding for identity expression. Alumni football provides the ideal context for this translation: a shared alumni affiliation accelerates the formation of trust, fostering profound emotional bonds (Evans et al., 2013) that are critical for mitigating loneliness (Cruwys et al., 2014). Consequently, athletic identity functions fundamentally as a bridge to social connectedness, indirectly bolstering mental wellbeing through this mediating mechanism (Clark et al., 2025; Antoniak et al., 2022).

Current research on adult sport participation has predominantly concentrated on the direct benefits of physical activity volume or the identity crises of elite athletes retiring from professional sport. However, this perspective often overlooks how amateur adults utilize specific social vehicles, such as alumni networks, to renegotiate their athletic identities in the long term. Consequently, the specific mechanisms of how post-participation sport involvement influences mental wellbeing through the interplay of identity and connectedness remain insufficiently explored.

In summary, based on this emerging social phenomenon of alumni football, this study attempts to deeply analyze its mechanism of impact on the mental wellbeing of adults from the perspective of social psychology. To clarify the distinction of this specific sporting context and its psychological pathways, this study proposes the following Research Questions (RQs):

- **RQ1:** Does alumni football participation directly predict mental wellbeing in adults?
- **RQ2:** Does athletic identity act as a standalone predictor of wellbeing, or does it carry potential limitations when not supported by social factors?
- **RQ3:** Do athletic identity and social connectedness form a serial mediation pathway?

By answering these questions, this study aims to enrich the explanatory framework of adult psychology theoretically and provide cases for universities and social organizations to utilize alumni network resources for mental wellbeing intervention and build supportive sports communities.

## 2 Research hypotheses and model construction

To clarify the psychosocial mechanisms through which alumni football participation influences adults' mental wellbeing, this study integrates self-perception theory and social identity theory to establish a comprehensive theoretical framework. Self-perception theory posits that individuals gradually form stable self-concepts through continuous observation of their own behaviors, whereas social identity theory emphasizes that once individuals internalize a group identity, their psychological states are shaped through social interaction and emotional bonding. Based on these complementary perspectives, this study develops a chain mediation model to explicate the pathways linking the key variables.

### 2.1 Direct relationship between alumni football participation and mental wellbeing

A substantial body of research demonstrates a positive association between physical activity and mental wellbeing outcomes (Chekroud et al., 2018). From a psychophysiological perspective, regular exercise alleviates anxiety and depressive symptoms by improving neuroendocrine functioning and reducing stress-related hormones. Alumni football combines relatively high physical intensity with recreational characteristics, not only activating physical functions but also providing adults with opportunities to temporarily disengage from occupational and daily stressors (Eime et al., 2013). Higher participation frequency is therefore associated with greater opportunities for emotional release and positive affective experiences. Accordingly, alumni football participation itself serves as an important predictor of mental wellbeing. Thus, the following hypothesis is proposed:

H1: Alumni football participation is positively associated with athletic identity.

### 2.2 The mediating role of athletic identity

According to self-perception theory, individuals infer their attitudes and identities by interpreting repeated behavioral patterns (Bem, 1972). When adults consistently engage in alumni football training and competitions, this stable behavioral involvement is gradually internalized into their self-concept, leading to the formation of an athletic identity such as perceiving oneself as a “football player” or a “team member” (Brewer et al., 1993). A positive identity constitutes an important psychological resource, enhancing self-esteem and perceived self-efficacy. For adults experiencing occupational stress or role conflict, an athletic identity beyond the work domain provides an additional source of self-worth, thereby contributing to improved mental wellbeing (Ronkainen et al., 2016). Based on this reasoning, the following hypotheses are formulated:

H2: Alumni football participation is positively associated with social connectedness.
H5: Athletic identity is positively associated with mental wellbeing.

### 2.3 The mediating role of social connectedness

Alumni football is inherently an interaction-based team sport. Unlike individual physical activities, football requires frequent verbal communication, tactical coordination, and physical contact among team members, creating favorable conditions for the development of social connectedness. As participation frequency increases, both the depth and breadth of interactions with teammates expand, facilitating the formation of close interpersonal ties. Social support theory suggests that high-quality social connectedness serve as a protective factor for mental wellbeing (Cohen and Wills, 1985). Within alumni football teams, teammates often function not only as sport partners but also as sources of emotional support, fostering a sense of belonging and acceptance that buffers life stress and enhances subjective wellbeing (Stevens et al., 2017). Accordingly, the following hypotheses are proposed:

H3: Alumni football participation is positively associated with mental wellbeing.
H6: Social connectedness is positively associated with mental wellbeing.

### 2.4 Chain mediating effects of athletic identity and social connectedness

Building on the preceding hypotheses, this study further examines the relationship between athletic identity and social connectedness. Theoretically, the formation of a collective identity serves as the cognitive foundation for social connectedness. Social identity theory posits that trust, cooperation, and cohesion among group members are grounded in shared identities (Ashforth and Mael, 1989); specifically, cognitive self-categorization (identifying as a group member) acts as a prerequisite for developing deep emotional attachments and group belongingness. In the context of alumni football, the dual identities of “alumni” and “teammate” substantially reduce interpersonal barriers. Stronger identification with the team is associated with higher levels of trust and emotional investment in teammates, as well as greater willingness to engage in proactive interaction and self-disclosure, which in turn relates to deeper social connectedness (Rees et al., 2015; Haslam et al., 2009). Consequently, our model proposes a continuous pathway where participation relates to identity, identity correlates with social bonds, and social bonds are linked to mental wellbeing. Based on this logic, the following hypotheses are proposed:

H4: Athletic identity is positively associated with social connectedness.
H7: Alumni football participation is indirectly associated with mental wellbeing through a serial mediation pathway involving athletic identity and social connectedness.

### 2.5 Serial mediation model construction

**Figure 1:**
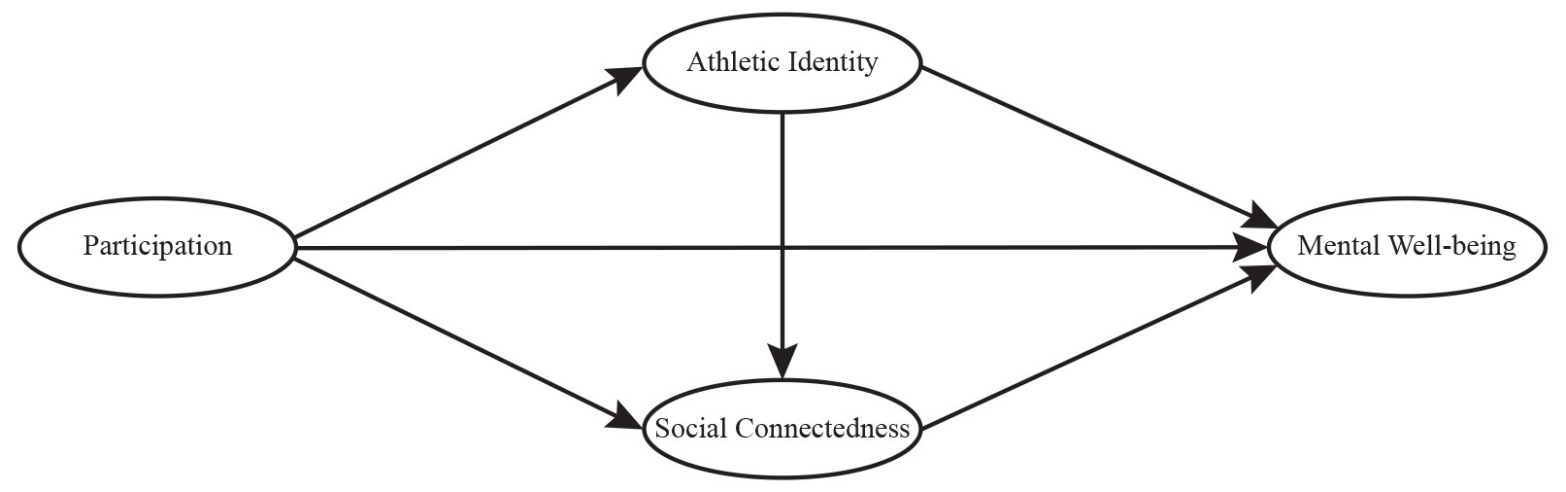
Conceptual model diagram.

Based on the theoretical analysis and hypotheses above, this study constructs a chain mediation model with alumni football participation as the independent variable and mental wellbeing as the dependent variable, as illustrated in Figure 1. Athletic identity and social connectedness are specified as sequential mediators to elucidate the internal mechanisms through which participation influences mental wellbeing. In addition, age, gender, and years of participation are included as control variables to account for potential confounding effects and to enhance the validity of the findings.

## 3 Methodology

### 3.1 Participants

This study adopts a cross-sectional questionnaire survey design targeting adult members of alumni football teams. The sampling frame primarily covers alumni football teams based in Wuhan, China. Leveraging the researcher's role as a coordinator for the regional university alumni football league, a convenience sampling strategy is employed. The online survey link is distributed via team-specific WeChat groups, which serve as the primary communication hubs for organization and interaction. To ensure the validity of the sample, the following inclusion criteria apply: (1) participants must be graduated adults to accurately reflect the “alumni” demographic; (2) they must have participated in alumni football activities at least once in the past month to ensure active engagement. All participants review and confirm an electronic informed consent form, indicating their understanding of the study purpose, the principle of anonymous data handling, and their right to withdraw from the study at any time. The questionnaire requires approximately 5-8 minutes to complete. A total of 265 questionnaires are returned. After applying predefined data screening criteria and excluding invalid responses, 240 valid questionnaires are retained for analysis, resulting in an effective response rate of 90.6 %. The sample is predominantly male, with a mean age of 33.4 years and an average football participation duration of 4.8 years.

### 3.2 Measures

The measurement instruments are developed based on established scales reported by (Gill et al. 2013), (Brewer et al. 1993), Lee and Robbins (1995), and (Tennant et al. 2007), with modifications to reflect the context of alumni football participation in China. Specifically, the measurement of alumni football participation is operationalized via frequency, intensity, and persistence, aligning with the multi-dimensional framework of behavioral engagement (Caspersen and Christenson, 1985; Bull et al., 2020). To enhance the suitability of the instruments for the specific cultural and research setting, all items undergo a standard translation-back-translation procedure, followed by semantic and contextual adaptation (Brislin and R., 1970). The final questionnaire consists of 18 items measuring four core constructs: alumni football participation (3 items), athletic identity (5 items), social connectedness (5 items), and mental wellbeing (5 items). All items are rated on a five-point Likert scale ranging from 1 (“strongly disagree”) to 5 (“strongly agree”), with higher scores indicating higher levels of the corresponding construct. In addition, demographic characteristics and years of football participation are collected and included as control variables in subsequent analyses. Reliability analysis indicates satisfactory internal consistency for all scales, with Cronbach's α coefficients ranging from 0.789 to 0.829. Regarding convergent validity, although the Average Variance Extracted values for athletic identity and social connectedness fall slightly below the 0.50 threshold, the Composite Reliability for all constructs exceeds 0.79. According to (Fornell and Larcker 1981), convergent validity remains adequate if the Average Variance Extracted (AVE) is less than 0.5 provided that Composite Reliability is higher than 0.6. Furthermore, the Fornell-Larcker criterion supports discriminant validity. As Table 1 shows, the square root of the AVE for each construct, which appears in the diagonal, exceeds its highest correlation with any other construct, indicating that the constructs are statistically distinct.

**Table 1:** Descriptive statistics, reliability, validity, and correlations.

**Variables**	**Mean**	**SD**	**α**	**AVE**	**CR**	**1**	**2**	**3**	**4**	**5**	**6**	**7**
Participation	3.20	0.81	0.789	0.564	0.793	0.751						
Athletic identity	3.77	0.63	0.796	0.443	0.797	0.529^**^	0.666					
Social connectedness	3.89	0.69	0.797	0.442	0.798	0.553^**^	0.476^**^	0.665				
Mental wellbeing	3.70	0.71	0.829	0.493	0.829	0.469^**^	0.382^**^	0.503^**^	0.702			
Age	33.4	5.2	–	–	–	−0.06	−0.01	−0.06	−0.12	**1**		
Gender	–	–	–	–	–	0.05	0.08	0.01	0.10	−0.06	**1**	
Years played	4.8	2.1	–	–	–	0.03	−0.03	0.11	−0.02	0.36^**^	0.18^*^	**1**

*N* = 240. AVE, Average Variance Extracted; CR, Composite Reliability. Values on the diagonal represent the square root of the AVE.

^**^p < 0.01, ^*^p < 0.05.

### 3.3 Data analysis

Data processing and analysis use SPSS 26.0 and AMOS 24.0 software. Prior to formal analysis, data screening ensures the absence of missing values as the online survey platform mandates responses to all items. The study calculates composite mean scores for each construct to represent the variables in subsequent analyses. Descriptive statistical analysis and Pearson correlation analysis among variables are primarily performed in SPSS. Subsequently, AMOS is utilized to execute Confirmatory Factor Analysis (CFA), validating the reliability and validity of the measurement model by calculating Composite Reliability (CR) and AVE. On this basis, a SEM is constructed to test the path relationships of the research hypotheses, and the bias-corrected percentile Bootstrap method is adopted to verify the significance of mediation effects.

## 4 Result

### 4.1 Descriptive statistics and correlations

The descriptive statistics, reliability coefficients, validity indices, and pearson correlation coefficients for all study variables are shown in Table 1. The results indicate that alumni football participation is significantly and positively correlated with athletic identity (*r* = 0.529, *p* < 0.01), social connectedness (*r* = 0.553, *p* < 0.01), and mental wellbeing (*r* = 0.469, *p* < 0.01). Furthermore, significant associations exist between the mediating variables and the outcome variable: athletic identity is positively correlated with social connectedness (*r* = 0.476, *p* < 0.01) and mental wellbeing (*r* = 0.382, *p* < 0.01), while social connectedness demonstrates a strong correlation with mental wellbeing (*r* = 0.503, *p* < 0.01). In contrast, the demographic control variables exhibit weak or non-significant correlations with the main psychological variables. These significant correlations among the core constructs provide preliminary data support for the subsequent model hypothesis testing.

### 4.2 Measurement model assessment

The CFA results indicate that the measurement model exhibits an excellent fit to the data: χ^2^ = 117.105, χ^2^/*df* = 0.908, *GFI* = 0.949, *AGFI* = 0.932, and *RMSEA* = 0.008. As shown in Table 2, the standardized factor loadings for all items ranged from 0.57 to 0.85 and are statistically significant (*p* < 0.001). These results indicate that the observed items effectively reflect their underlying constructs. Coupled with the reliability and validity indices reported in Table 1, the measurement model demonstrates adequate convergent validity and internal consistency.

**Table 2:** Factor loadings of the measurement model.

**Construct**	**Item**	**Factor loading**
Participation	Q4: The frequency of my participation in alumni football activities.	0.854
	Q5: My exercise intensity during alumni football activities is usually high.	0.745
	Q6: I persist in attending scheduled team activities regardless of how busy I am.	0.639
Athletic identity	Q7: I consider myself an alumni football player.	0.718
	Q8: Football is an indispensable part of my life.	0.605
	Q9: People who know me consider me a football enthusiast.	0.704
	Q10: Participating in alumni football makes me feel proud.	0.719
	Q11: I feel lost or uncomfortable when I cannot play football.	0.565
Social connectedness	Q12: I can feel emotional support from others during alumni football activities.	0.662
	Q13: Alumni football gives me a strong sense of group belonging.	0.679
	Q14: I am willing to seek support from teammates when encountering difficulties.	0.695
	Q15: I trust and am willing to rely on other members of the team.	0.634
	Q16: Alumni football activities provide me with frequent opportunities for social interaction.	0.651
Mental wellbeing	Q17: I feel useful and able to realize my self-worth.	0.694
	Q18: I feel capable of handling problems in life well.	0.730
	Q19: Recently, I have felt happy and relaxed.	0.665
	Q20: I am satisfied with my current life.	0.715
	Q21: I hold a positive and optimistic attitude toward the future.	0.705

### 4.3 Structural model assessment

**Figure 2:**
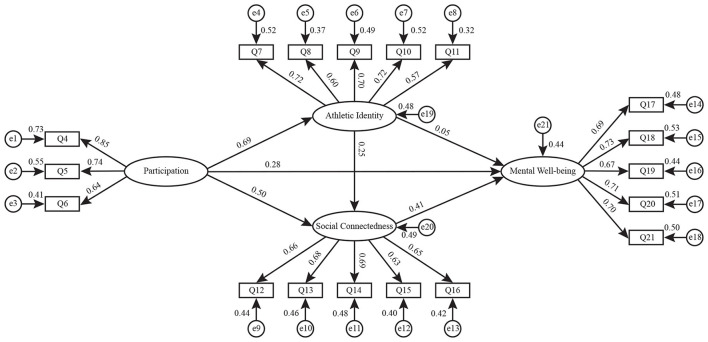
Structural equation model with standardized path coefficients.

Following the confirmation of the reliability and validity of the measurement model, the structural equation model tests the hypothesized pathways. The structural model demonstrates a good fit to the data, as illustrated in Figure 2. As presented in Table 3, the results indicate that alumni football participation is positively associated with athletic identity (β = 0.69, *p* < 0.001), social connectedness (β = 0.50, *p* < 0.001), and mental wellbeing (β = 0.28, *p* < 0.05), supporting H1, H2, and H3. Regarding internal mechanisms, athletic identity is positively associated with social connectedness (β = 0.25, *p* < 0.05), supporting H4. Social connectedness shows a significant positive association with mental wellbeing (β = 0.41, *p* < 0.001), supporting H6. Conversely, the direct path from athletic identity to mental wellbeing is not statistically significant (β = 0.05, *p*>0.05); thus, H5 is not supported. In terms of relative strength, participation exhibits the strongest association with athletic identity among all observed direct paths.

**Table 3:** Path coefficients and hypothesis testing results.

**Hypothesis**	**Structural path**			**Std. β**	**S.E**.	**C.R**.	**Result**
H1	Participation	→	Mental wellbeing	0.28^*^	0.134	2.209	Supported
H2	Participation	→	Athletic identity	0.69^***^	0.096	7.024	Supported
H3	Participation	→	Social connectedness	0.50^***^	0.117	4.182	Supported
H4	Athletic identity	→	Social connectedness	0.25^*^	0.112	2.286	Supported
H5	Athletic identity	→	Mental wellbeing	0.05	0.120	0.430	**Not supported**
H6	Social connectedness	→	Mental wellbeing	0.41^***^	0.125	3.529	Supported

Std. β = Standardized path coefficient; S.E., Standard Error; C.R., Critical Ratio.

^***^p < 0.001, ^*^p < 0.05.

Bold values highlight the non-significant direct relationship between athletic identity and mental wellbeing (H5).

### 4.4 Mediation analysis

To examine the mediating mechanisms, the bias-corrected percentile bootstrapping method (with 5,000 resamples and a 95% confidence interval) is employed to test the significance of the indirect effects. The full structural model demonstrates satisfactory fit indices (χ^2^/*df* = 0.908, *CFI* = 1.000, *RMSEA* = 0.001). The results, summarized in Table 4, indicate that the total indirect effect of alumni football participation on mental wellbeing is statistically significant (Estimate = 0.341, 95% CI = [0.103, 0.593]). Since the confidence interval does not include zero, this confirms that the mediators effectively transmit the influence of participation to mental wellbeing.

**Table 4:** Bootstrap analysis of mediation effects.

**Mediation path**	**Point**	**95% confidence interval**		**Conclusion**
	**Estimate**	**Lower**	**Upper**	
**Total indirect effect**	**0.328**	**0.103**	**0.593**	**Significant**
**Specific Indirect Effects**				
Ind1: Participation → identity → wellbeing	0.035	−0.155	0.212	Not significant
Ind2: Participation → connectedness → wellbeing	0.217	0.083	0.428	Significant
**Ind3: Participation** **→** **Identity** **→** **Connectedness** **→** **Wellbeing**	0.076	0.020	0.253	**Significant (H7)**

Bootstrap sample size = 5,000. CI, Bias-corrected confidence interval.

Bold values highlight the significant serial mediation path (H7).

Regarding the specific pathways, the indirect effect mediated solely through athletic identity is not statistically significant (Ind1, Estimate = 0.035, 95% CI = [−0.155, 0.212]), which aligns with the non-significant direct relationship between identity and wellbeing observed in the structural model. In contrast, the path mediated via social connectedness emerges as the strongest specific indirect effect (Ind2, Estimate = 0.217, 95% CI = [0.083, 0.428]), highlighting social connectedness as a strongest specific path for psychological benefits. Crucially, the hypothesized chain mediation path (Participation → Athletic identity → Social connectedness → Mental wellbeing) is statistically significant (Ind3, Estimate = 0.076, 95% CI = [0.020, 0.253]). Comparing the specific indirect effects, the pathway via social connectedness (Ind2) presents a larger effect size than the serial pathway (Ind3), while the pathway via athletic identity alone (Ind1) effectively contributes strictly through the serial sequence.

## 5 Discussion

This study employs a structural equation modeling approach to examine the mechanisms through which alumni football participation associates with adults' mental wellbeing. The findings indicate that the associations do not operate through a single pathway. Instead, athletic identity and social connectedness operate in sequence, forming a multi-layered chain mediation structure. The following discussion focuses on the main theoretical mechanisms, clarifies the psychological transition from individual identity formation to group-based social bonding, and considers practical implications for alumni sports initiatives alongside brief reflections on study limitations.

### 5.1 Interpretation of results

The results confirm that alumni football participation significantly and positively correlates with mental wellbeing, aligning with prior evidence on the psychological benefits of team-based physical activity. As a sport characterized by both relatively high physical intensity and a strong collective nature, alumni football associates with emotional regulation not only through physiological mechanisms but also by offering adults a temporary psychological distance from occupational and daily stressors (Singh et al., 2023). These observations theoretically suggest that sustained engagement in football remains a relevant correlate of mental wellbeing many years after graduation (Graupensperger et al., 2020).

Further path analyses underscore the central role of social connectedness within the overall mechanism. Social connectedness independently mediates the relationship between participation and mental wellbeing, presenting the largest effect size among all indirect pathways. This pattern indicates that the psychological benefits of alumni football align closely with its social and relational qualities rather than deriving solely from physical exertion (Eather et al., 2023). Through repeated training and competition, participants gradually develop stable and meaningful interpersonal ties within the team. Such experiences of belonging and emotional support play an important role in alleviating feelings of loneliness and enhancing life satisfaction. From an empirical perspective, these findings align with social capital theory, which views sport settings as important arenas for adults to accumulate social resources and access emotional support (Zhou and Kaplanidou, 2018).

In contrast, the role of athletic identity in the mental wellbeing process appears more nuanced. The direct association between athletic identity and mental wellbeing lacks statistical significance, suggesting that merely perceiving oneself as a team member does not automatically correlate with higher psychological wellbeing. However, the significant chain mediation clarifies the functional position of athletic identity in this process. Rather than acting as a direct source of mental wellbeing benefits, athletic identity operates by facilitating social engagement. Individuals with stronger athletic identity tend to invest more actively in team interactions, develop greater emotional attachment to the group, and ultimately experience better mental wellbeing through strengthened social connectedness. These results indicate that identity contributes to psychological outcomes only when it embeds in sustained and meaningful social relationships.

### 5.2 The transformation mechanism from athletic identity to social capital

In terms of theoretical application, this study extends the explanatory boundaries of Social Identity Theory within the adult alumni population. Traditional research often emphasizes the direct protective association of identity with mental health; however, the data from this study indicate that in adulthood, following detachment from the campus environment, a mere identity label is insufficient to directly correlate with psychological benefits. This study proposes that in this context, athletic identity primarily functions as a mechanism of Social Entry (Schmid et al., 2025). It provides alumni with a common language and symbolic representation, thereby lowering the threshold for interpersonal interaction. This identity cognition serves as the psychological prerequisite for integrating into the group rather than the ultimate goal; it requires activation through substantive interactive behaviors to realize its function in promoting psychological wellbeing (Stevens et al., 2024).

Simultaneously, the establishment of the chain mediation model further confirms that the sports arena is a critical space for adults to accumulate social capital. The complete mediation effect clearly depicts the dynamic process from individual psychological construction to interpersonal resource transformation. This substantiates that the core value of alumni football lies in the reproduction of social relations. Through regular sports participation, alumni translate abstract athletic identity into concrete social connectedness, such as trust, reciprocity, and emotional support. This finding enriches the contextual application of Social Capital Theory, revealing how alumni sports provide individuals with social resources to cope with psychological stress through an identity-to-capital transformation mechanism.

### 5.3 Building a dual-driven model for alumni sports

Based on the theoretically driven framework and statistically consistent paths, the findings offer potential directions for future practical validation. Rather than serving as firm prescriptions, these associative patterns suggest that alumni organizations and relevant managers explore shifting from a traditional mindset focused solely on competitive outcomes to a dual-driven model of competition and socialization.

On one hand, managers emphasize activity designs that foster friendship through football to maximize the social dividends of sports activities. Given the strong association of social connectedness with mental wellbeing, the organization of alumni football events extends beyond on-field competition to focus on building off-field communication platforms (Shu, 2024). It is recommended to institutionalize post-match social gatherings and exchange meetings alongside traditional match formats, and to encourage mixed-team matches across different grades and majors. These measures aim to create high-frequency, in-depth interaction opportunities, helping alumni extend on-field comradeship into an off-field social support network.

On the other hand, it is necessary to prioritize the role of team culture in shaping athletic identity, treating it as the cornerstone for establishing social connections. Although athletic identity does not directly correlate with mental wellbeing, it serves as a necessary antecedent for establishing social connections. Therefore, managers strengthen alumni identification with the dual identity of player and alumnus by designing unified visual identity systems, establishing team honor systems, and holding regular induction ceremonies. By reinforcing identity to stimulate a sense of belonging, managers guide individuals to actively cross social thresholds and establish substantive social connections.

Crucially, stakeholders integrate these practical interventions while acknowledging the study limitations. The cross-sectional nature of the data reveals associative patterns rather than strict causal directions. Therefore, organizers implement these community-building strategies while simultaneously monitoring longitudinal outcomes to evaluate the long-term psychological benefits.

### 5.4 Limitations and future research directions

Although this study reveals the chain mediation mechanism by which alumni football participation associates with mental wellbeing, certain limitations exist due to objective constraints.

First, the cross-sectional nature of the research design limits causal inference. This study validates model pathways based on data from a single time point, making it difficult to fully confirm the causal direction between variables. Future research benefits from employing longitudinal designs or experimental intervention designs to further verify the dynamic causal directions between participation behavior and mental health.

Second, the representativeness of the sample requires improvement. Due to the sampling scope and the characteristics of football participation, the sample consists predominantly of male participants from Chinese alumni networks. This potentially limits the generalizability of the findings to female alumni, participants from different cultural backgrounds, or other types of sports. Future research should aim to validate this model in diverse cultural contexts to explore whether the mechanism of alumni bonding differs across societies, as well as expanding sample diversity to explore differences across genders.

Third, data collection relies mainly on self-reported questionnaires. Despite control measures, the data remain susceptible to subjective factors. Future research incorporates more objective measurement indicators or combines qualitative interview methods to enhance data richness and the robustness of the conclusions.

## 6 Conclusion

This study constructs and validates a chain mediation model to demonstrate that alumni football participation positively associates with adult mental wellbeing. The process features a dynamic transition from identity construction to social capital transformation. The findings reveal that although athletic identity does not directly correlate with mental wellbeing, it functions as a necessary antecedent for establishing social connectedness. By strengthening the sense of team belonging, athletic identity facilitates high-quality social interactions that subsequently relate to psychological benefits. This pattern indicates that social connectedness occupies a core position within this psychological framework. Alumni football serves as a composite activity possessing both identity verification and social bonding functions, and its core value lies in the construction of a robust social support network through shared athletic engagement. Given the cross-sectional design, the current findings establish an associative framework rather than a definitive causal sequence. Consequently, the proposed practical initiatives serve as potential strategies for community wellbeing rather than guaranteed psychological interventions. Building upon these limitations, future research utilizes longitudinal designs to track how specific social interactions during alumni sports dynamically shape mental health outcomes over time.

## Data Availability

The original contributions presented in the study are included in the article/supplementary material, further inquiries can be directed to the corresponding author.
